# Ureteroiliac fistula after oncological surgery: Case report and review of the literature

**DOI:** 10.1515/med-2022-0588

**Published:** 2022-12-12

**Authors:** Ettore Mearini, Alessio Paladini, Valerio Cellini, Matteo Mearini, Graziano Felici, Andrea Vitale, Giovanni Cochetti

**Affiliations:** Department of Medicine and Surgery, Division of Urology, University of Perugia, Perugia, 06129, Italy

**Keywords:** ureteroiliac fistula, haemorrhagic shock, emergency treatment

## Abstract

Fistulas arising between ureters and iliac arteries (UAF) are rare pathological events and frequently require emergency treatment, as they are associated with massive haematuria and haemorrhagic shock. The medical history plays a key role in the diagnostic and therapeutic process, as it allows to include UAF among the differential diagnoses of gross haematuria. The emergency treatments of fistulas arising between the urinary system and the vascular system include the *open* repairing surgery or the endovascular grafting, the latter generally better tolerated by patients suffering from multiple comorbidities or not eligible for traditional surgery. Nephrostomy or ureteral stent can be used to drain the affected upper urinary tract temporarily or permanently. Herein, we reported two cases of oncological patients affected by UAF and treated successfully by endovascular procedures. Furthermore, we performed a narrative review of the literature concerning UAF and its diagnostic and therapeutic management. Although our study did not allow us to state definitive conclusion about the diagnostic and therapeutic management of UAF due to small sample size, our findings support previous experiences in favour of the treatment of fistulas with an endovascular approach.

## 1 Introduction

Fistulas arising between ureters and iliac arteries (UAF) are rare pathological events [1] and frequently require emergency treatment, as they are associated with massive haematuria and haemorrhagic shock, with an overall mortality ranging between 7 and 23% [2]. Over the years, many pathogenetic factors of fistulization have been detected, such as arterial atherosclerosis, pelvic and retroperitoneal surgery (oncological and vascular), radiation therapies, and indwelling ureteral stenting [1,3,4,5,6,7,8]. The emergency treatments of fistulas arising between the urinary system and the vascular system include the *open* repairing surgery or the endovascular grafting, the latter generally better tolerated by patients suffering from multiple comorbidities or not eligible for traditional surgery [3,4]. Nephrostomy or ureteral stent can be used to drain the affected upper urinary tract temporarily or permanently.

**Figure 1:**
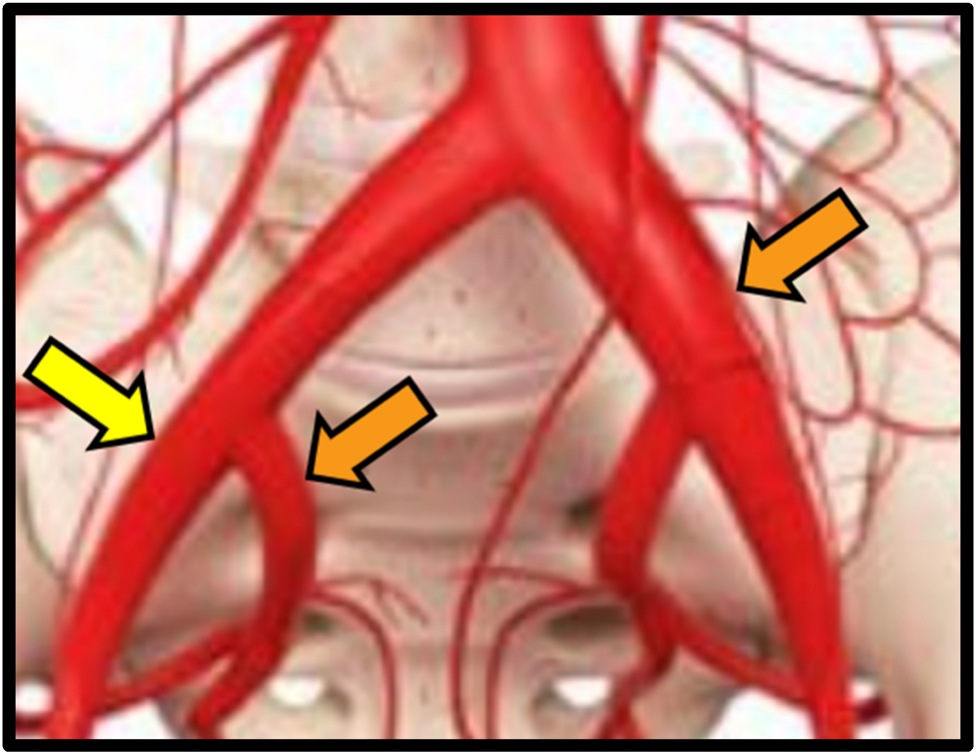
Site of UAF. Yellow dart: case 1 (proximal external iliac artery). Orange darts: case 2 (distal left common iliac artery and proximal left hypogastric artery).

Herein, we reported two cases of oncological patients treated for UAF, one of them affected by bilateral fistula. Secondary aim was to perform a narrative review of the literature concerning UAF and its diagnostic and therapeutic management (Figure 1).

Table 1 shows the main demographic and clinical data.

**Table 1:** Clinical history of our patients

	Case 1	Case 2	
	1st episode	1st episode	2nd episode
Gender	Male	Female	
Age at UAF onset	72	56	64
Smoking	Yes	No	No
Comorbidities and history	Aorto-iliac atherosclerosis	Comorbidities: AH, CKD, chronic anaemia. History: vasculitis, left iliac-femoral DVT.	
Charlson comorbidity index (CCI)	9	4	5
Previous surgery (abdominal, retroperitoneal)	None	Left nephrectomy (February 2014) for chronic erosive pyelitis	
Previous Surgery (oncological)	RARC with lymphadenectomy	Hystero-adnexectomy + lymphadenectomy	
Urinary diversion	Ileal orthotopic neobladder	none	
Chemotherapy (CHT) – immunotherapy (IO)	Adjuvant CHT + IO	Adjuvant CHT	
Radiotherapy	No	Yes (pelvic)	
Periodical stent substitution	Yes (right)	Yes, bilateral (after RN, only right ureteral substitutions)	Yes (right ureter)
Stenting placement: indication	Hydronephrosis (right ureter-neobladder anastomosis stenosis) and right pyelonephritis	Hydronephrosis (ureteral stenosis)	
Side/level fistula	Right proximal EIA	Left CIA (residual ureter)	Right proximal IIA
Indwelling stent at UAF diagnosis	Yes	No	Yes
First CT scan	Positive	Negative	Not performed
Open surgery	No	No	No
Endovascular approach	Yes	Yes	Yes
Angiography/aortography	Positive	Positive	1st negative → 2nd positive
Pyelography	Positive	Positive	Not performed
Ureteropyeloscopy	Not performed	Performed – not diagnostic for massive bleeding	Performed – not diagnostic for massive bleeding
UAF onset after first stenting	16 months (February 2020)	7 years (May 2014)	13 years (July 2019)
Arterial embolization	Right hypogastric artery	Left hypogastric artery	Right hypogastric artery
Materials and type of arterial by-pass	Viabahn^®^ 5 mm × 100 mm between right common iliac artery and External iliac artery	Viabahn^®^ 9 mm × 100 mm in left Common iliac artery	Viabahn^®^ 16 mm × 12 mm × 100 mm between right proximal common iliac artery and External iliac artery
Ureteral embolization	No	Yes	No
UAF onset after RT (months)	/	Unknown	Unknown
Stent material and Ch	Polyurethane hydrophilic-coated double J 8 Ch	nd	Allium^®^, Silicone hydrophilic-coated (Vortek^®^) double J 7 Ch
Rebleeding	No	No	Yes
Indwelling ureteral stent/nephrostomy post UAF	Nephrostomy	Right ureteral stent	Right ureteral stent
Therapy at discharge	Acetilsalicilic acid 100 mg	LMWH and antibiotics	LMWH and antibiotics
Follow-up (complications)	No	No	Rebleeding; right uretero-colic fistula
Follow-up (further surgery/endovascular surgery)	No	No	Endovascular prothesis placement; haemicolectomy and colostomy
Death	3 months earlier for BC metastatic progression	No	No

AH: arterial hypertension; CDK: chronic kidney failure; DVT: deep venous thrombosis; RARC: robot-assisted radical cystectomy; EIA: external iliac artery; CIA: common iliac artery; IIA: internal iliac artery; LMWH: low molecular weight heparin; BC: bladder cancer.

## 2 Case 1

**Figure 2:**
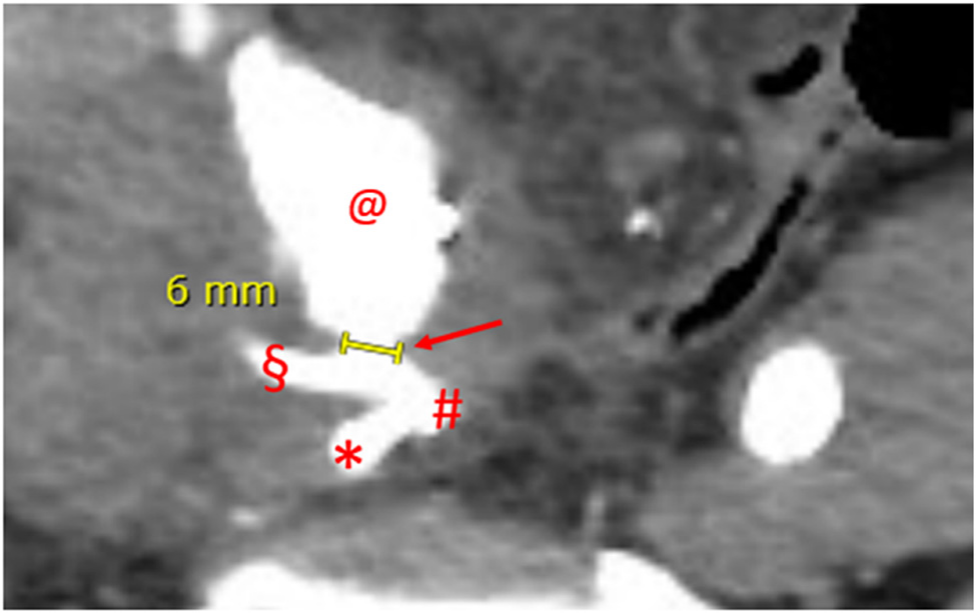
Angio-CT, axial plane, arterial phase, case 1. Major diameter of the UAF. Dart: UAF with concomitant iodinated contrast leakage; #: common right iliac artery bifurcation; *: hypogastric artery; §: external iliac artery; and @: pseudoaneurysm sac.

**Figure 3:**
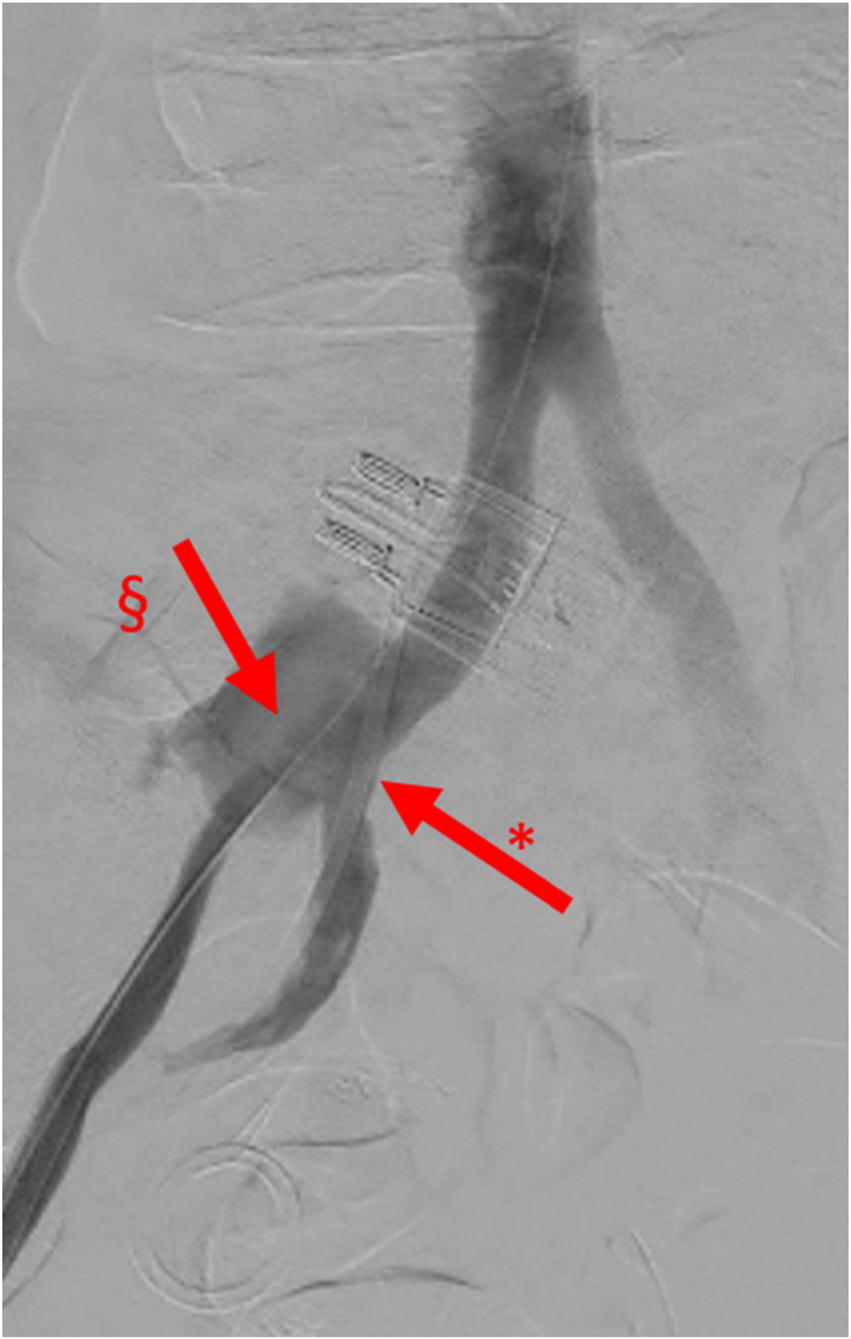
Angiography, image with no digital subtraction, frontal view, late arterial phase, case 1. Iodinated contrast leakage from UAF. *: right ureteral double J stent and §: contrast leakage from UAF.

Caucasian 72-year-old male, smoker, suffering from moderate and diffuse atherosclerosis along the aortic-bis-iliac and iliac bifurcations, underwent robot-assisted radical cystectomy (RARC) with orthotopic ileal neobladder reconstruction for muscle invasive high-grade urothelial carcinoma of the bladder cT2N2M0 on October 2018. After the onset of pulmonary metastases, he underwent adjuvant chemo-immunotherapy. Eleven months after RARC, an 8 Charrière (Ch) stent was placed for slight right hydronephrosis onset due to benign stenosis of the neobladder-ureteral anastomosis. The patient, not being a candidate for major corrective surgery of stenosis due to systemic progression of the muscle invasive bladder cancer, underwent one double J ureteral stent replacement for palliative purposes. The patient was re-admitted to our clinic for urosepsis showing hyperpyrexia (body temperature >39°C) associated with shiver and a slightly increased right hydronephrosis compared to the previous condition, with the impairment of renal function (creatinine and estimated glomerular filtration rate were 2.18 mg/dl and 31.64 mL/min, respectively) despite the double J stent resulted correctly placed. The patient was treated by empiric broad-spectrum antibiotic therapy and supportive therapy with quick recovery from all symptoms. On the fifth day of hospitalization and close to discharge, a lipothymic episode and stinging pain in the right iliac fossa with massive haematuria occurred, determining severe shock. Haemoglobin level dropped from 9.1 to 7.4 g/dL in few hours, and emergency blood transfusion was started. An emergency contrast-enhanced total body computed tomography (CT) scan was performed, showing a spread of contrast from a 6 mm injury of the right external iliac artery close to ureteral cross. The patient underwent subsequent transfemoral arteriography that revealed a pseudoaneurysm of the proximal right external iliac artery. The embolization of the right internal iliac artery using flakes of polyvinyl alcohol, and the placement of a 100 mm × 5 mm stent-graft (proximal-end in the common iliac artery, distal-end in the external iliac artery), allowed the prompt resolution of the haemorrhage without further complications. Along the day of the emergency, a total of five blood transfusions were administered consecutively, and another transfusion was made the day after. The subsequent antegrade pyelography confirmed the presence of contrast leakage near the external iliac artery, thus confirming the diagnosis of UAF. The ureteral stent was removed 16 days after the procedure and permanent right nephrostomy was placed simultaneously. The patient was discharged in general stationary conditions, with 1.63 mg/dL of creatinine levels and 10.3 g/dL of haemoglobin. The patient died of bladder cancer 3 months after the endovascular procedure without any other complications related to the fistula. The patient signed informed consent for anonymous data use and publication (Figures 2 and 3).

## 3 Case 2

**Figure 4:**
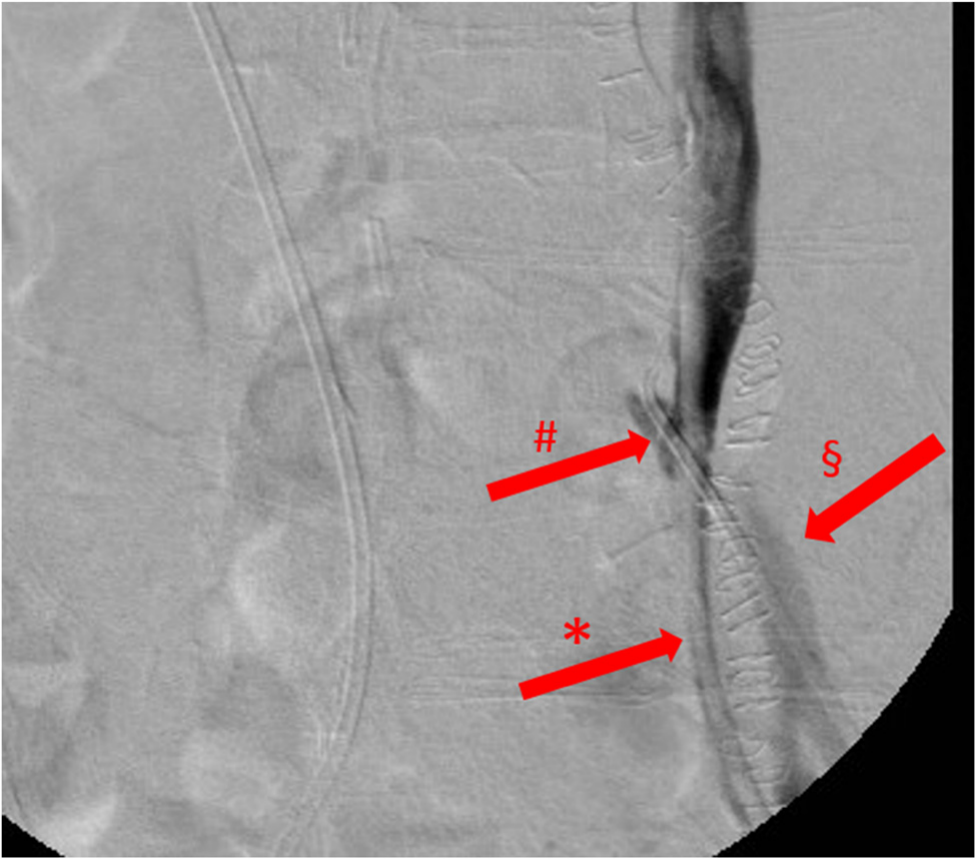
Retrograde ureterography, image with no digital subtraction, frontal view, case 2, first episode. Iodinated contrast leak from the left ureter into the distal left common iliac artery. *: left ureteral stent; §: left common iliac artery; and #: UAF.

A 56-year-old Caucasian woman underwent hystero-adnexectomy plus pelvic lymphadenectomy and adjuvant radio-chemotherapy treatment for squamous cell carcinoma of the uterine cervix in 2000. The patient, suffering from post-actinic retroperitoneal fibrosis and hydronephrosis was treated by bilateral ureteral stenting in 2007. In 2014, the patient underwent left radical nephrectomy (RN) for chronic pyelonephritis associated with severe haemorrhage. Few months after RN, the patient was re-admitted to our clinic for gross haematuria reaching a haemoglobin level of 6.6 g/dL, thus requiring blood transfusions. CT angiography and retrograde pyelography resulted negative for contrast leakage, so a bilateral ureteropyeloscopy and provocative retrograde pyelography were performed: the latter manoeuvre showed contrast leakage from the left ureteral stump into the iliac artery (Figure 4).

Ureteral embolization using a copolymer of ethylene and vinyl alcohol and percutaneous arterial stenting of the left common iliac artery by placement of 9 mm × 100 mm Viabahn^®^ stents were performed. After 8 years of good health in which ureteral stent was periodically substituted, gross haematuria with haemoglobin level at the admittance of 6.8 g/dL requiring immediate blood transfusions, fever, and pain in the right side of the abdomen occurred and the patient was re-admitted to our clinic. Cystoscopy showed right ureteral bleeding and the ureteroscopy confirmed the ureteral origin of the bleeding but was not diagnostic for bleeding cause, whereas the arteriography did not show any arterial leakage or other source of bleeding in urinary system. CT angiography was not performed for severe chronic renal failure. As the bleeding was persisting, the ureteropyeloscopy was repeated but resulted negative for haematuria. Subsequent arteriography showed leakage of contrast from the proximal right hypogastric artery. The embolization of the right hypogastric artery, and concomitant positioning of Viabahn® vascular endoprosthesis and a silicone hydrophilic-coated single J 7 Ch ureteral stent on already-in-place self-expanding elastic nitinol alloy covered by a unique polymer (Allium^®^), allowed the resolution of the haematuria with a rapid improvement in the general conditions of the patient. Haematuria recurred after 8 and 10 months because of vascular complications, so a double self-expanding metalized endovascular prosthesis for pseudoaneurysm of the right EIA and an aortic-bisiliac endovascular prosthesis due to the collapse of the right iliac stent and the onset of a 10 cm pseudoaneurysm were, respectively, placed. At the 13th month of follow-up, the onset of a ureteral-intestinal fistula required to perform a colostomy. Six months after, the ureteral double J stent was removed and an indwelling nephrostomy was placed. The patients is alive to date. The patient signed informed consent for anonymous data use and publication.

## 4 Discussion

The onset of UAF is rare event, which could lead to dramatic consequences. It should be suspected in patients suffering from undefined continuous or intermittent haematuria with specific risk factors. The clinical presentation can be extremely variable and imaging may not be diagnostic at first analysis [1]. Therefore, the detailed and accurate analysis of patient’s comorbidity is mandatory for a correct clinical work-up. Smoking, history of pelvic cancer, previous pelvic or retroperitoneal surgery and radiation therapies, atherosclerosis, or chronic indwelling ureteral stenting were proved being associated with higher risk of UAF [1,9,10,11] (Table 2). Abdominal and pelvic oncological surgery and vascular surgery were reported from 63.8 to 69.5% of patients with UAF and van den Bergh et al. highlighted that they were independent risk factors [1].

**Table 2:** Main risk factors reported to be associated with UAF

	van den Bergh et al. [1]	Batter et al. [9]	Das et al. [11]	Subiela et al. [10]
	139 patients	37 patients	118 patients	94 patients
Surgery	54% (oncologic) + 31% (vascular)	68%	69.5%	63.8%
RT	Not specified	46%	48.3%	57.4%
Ureteral stenting	Not specified	65%	73.7%	91.5%

Radiotherapy and ureteral stenting were proved being in 46–57.4 and 65–91.5% of patients suffered from UAF, respectively. It is to be noted that the incidence of both these risk factors has increased over the years. The explanation of these data could be related to the three possible causes: (1) the growing and progressive use of minimal invasive surgery, specifically endovascular and endourological ones; (2) the increasing application of RT in cancer therapeutic management; and (3) the ageing of the population so that over the years the proportion of elderly patients to be treated has increased. Therefore, the most innovative therapeutic strategies, mainly for pelvic oncological disease, paradoxically may favour UAF incidence [5,12,13,14].

In fact, from the pathogenetic point of view, pelvic RT and repeated ureteral manipulation favour the degeneration of the ureteral wall and the scar-adhesion reaction of the periureteral tissues. Chronic inflammation, which is stimulated and exacerbated by RT and ureteral stenting, promotes fibrogenesis, ischaemic vascular damage, tissue atrophy, and necrosis [1,15,16]. The damage of the sclerotic periureteral tissue is also favoured by the pulling effect of the pulsatility of the close iliac arteries [12,17]. Beside the ureteral damage, the weakening of the arterial wall should be considered as the consequence of radiotherapy, diabetes, systemic arterial hypertension, atherosclerotic disease, and *vasa vasorum* deficiency [2], which are all typical diseases of elderly. Therefore, a patient affected by the aforementioned risk factors should be considered a high risk for UAF development. Clinical feature of UAF is haematuria, massive or even intermittent, which is the most common symptom and is variously accompanied by flank pain and haemodynamic instability. Krambek et al. in 2005, in their diagnostic algorithm, considered haematuria, the presence of bladder clots, and flank pain the main symptomatological triad.

The most common symptoms reported in literature are summarized in Table 3.

**Table 3:** The most common symptoms of UAF (%)

		Krambek et al. [3] (8 patients)	van den Bergh [1] (139 patients)	Das et al. [11] (118 patients)	Subiela et al. [10] (94 patients)	Our cases (2 patients, 3 UAF)
Main symptoms (%)	Haematuria	100	74	100	100	3/3: 100%
	Flank pain	57	17	7.6	12.8	3/3: 100%
	Fever	n.r.	7	n.r.	n.r.	3/3: 100%
	Hydronephrosis	n.r.	n.r.	n.r.	26.6	2/3: 66%
	Haemodynamic instability	n.r.	n.r.	17.8	21.3	2/3: 66%

n.r.: not reported.

Fever was reported as not frequent symptom in literature [1,2], whereas it occurred in both our cases and it anticipated the massive haematuria. We believe it is a significant symptom because it could be premonitory of the onset of haematuria. This could be due to the complete fistulization between ureter and iliac artery that favours the passage of pathogens and pro-inflammatory factors from an infected and inflamed ureter into the bloodstream, leading to fever and urosepsis, as occurred in our first case [18]. Therefore, fever, especially if accompanied by macroscopic haematuria, in patients with risk factors, could be useful in directing the differential diagnosis of haematuria towards UAF. Imaging evaluation may benefit from angiography and contrast-enhanced CT scan, the latter not always appreciated from all authors in Literature. Krambek et al., in their diagnostic algorithm, did not include CT as the first-line examination for its low detection rate (50% of patients), recommending angiography and provocative angiography as the most useful diagnostic tools. However, in their study, CT scan was tested on only four patients, which are a too small sample to state a definitive recommendation [3]. van den Bergh et al. found similar data, reporting angiography and CT sensibility of 69 and 42%, respectively. The authors also showed ureter-pyelography sensibility of 52%, specifying how its diagnostic capacity depends on the speed of the blood flow out of the fistulized artery, asserting that a blood flow of ≥3 mL/s from the artery through the fistula into the ureter is unlikely to allow a pyelographic diagnosis. In addition, this study highlighted the need of performing an average of 2.4 radiological studies for a single patient before a definitive radiological diagnosis [1]. The diagnostic accuracy (%) of CT scan has been reported in some more recent studies as extremely variable, ranging from 0 to 55% [16,19,20].

Subiela et al. reported the number and type of diagnostic techniques used in the most recent studies from 1990 to 2017: the prevalence of angiography and angioTC employment was 36.2 and 29.8%, respectively, but the authors did not refer their diagnostic accuracy. However, the authors stated that CT scan with contrast was the most used and accurate method for the diagnosis of UAF [10]. In our first case, the indication for CT scan was mandatory due to the lipothymic-neurological signs that anticipated the severe haemorrhagic shock. CT scan in this case was diagnostic, and angiography only confirmed and targeted the fistula for the endovascular treatment. The antegrade pyelography carried out after endovascular treatment was purely descriptive. The positivity of all radiological exams is certainly attributable to the size of the fistula (6 mm in maximum diameter) which, in the acute phase of rupture, determined a blood outflow of about 7 mL/s, considering that usually the speed of blood flow at the level of the iliac artery is about 30 cm/s.

In our second case, ureteroscopy with retrograde pyelography was diagnostic for UAF, while CT was negative. When the UAF has risen again, the arteriography initially was negative, whereas resulted positive during combined endourological-endovascular surgery. CT scan in the second episode was not practiced since, in the concrete suspicion of a UAF and because of the patient’s severe renal failure, priority was given to the angiographic study, thus allowing performing simultaneously both diagnosis and treatment (Table 4).

**Table 4:** Diagnostic rate (%) of imaging

		Okada et al. [20] (11 patients)	van den Bergh et al. [1] (139 patients)	Das et al. [11] (118 patients)	Our case (2 patients, 3 UAF)
Imaging (% diagnostic)	CT scan	27	42	36	1/2 (50%)
	Angiography	45	69	72	2/3 (66%)
	UPS/RETROGRADE PYELOgraphy	Not performed	52	61	3/3 (100%)

UPS: ureteropyeloscopy.

Concerning therapeutic management, Krambeck et al. in 2005 suggested an algorithm that took into account being fit for surgery, reserving the endovascular approach only to patients not eligible for surgery or whatever with contraindications to it [3]. However, many studies reported that the endovascular approach is equally effective and better tolerated by the patients than traditional open surgery, with lower risks related to procedure [1,4,9,11,12,15,16,17]. In our experience, both the patients were treated by an endovascular approach, therefore through arterial embolization and the placement of endovascular prostheses and stents, and did not need open surgical correction. The endovascular approach, especially in the first patient, proved to be the fastest treatment in conditions of absolute emergency and haemodynamic instability, allowing the prompt resolution of the massive bleeding and the quick improvement of health conditions.

Subiela et al. reported post-procedural outcomes and mortality in patients undergoing vascular endoprosthesis or vascular stent implantation [10]. Overall post-procedural complications were 10%: early complications, defined as event within 30 days, occurred in 4.2% of patients and included femoral arterial thrombosis, wound infection, rebleeding, and graft-skin fistulization. Late complications, defined as event over 30 days, occurred in 17% of patients at a median follow-up of 8 months and included rebleeding (7.5%), endovascular stent thrombosis (3.2%), retroperitoneal abscess and urosepsis (2.1%), arterial thrombosis (1%), and limb claudication (1%). Overall mortality was 27.6% (26/94) at the follow-up, but only 2.1% died from complications related to UAF. Okada et al. in their case series of 11 patients evaluated complications and mortality related to the endovascular approach of UAF, describing no rebleeding at 1 and 2 years in 76.2 and 40.6% of patients, respectively, with only one death related to UAF [20]. In our experience, the first patient died from metastatic bladder cancer, and in the short 3-month follow-up, there were no UAF-related complications. The second patient is still alive despite she needed two endovascular re-operations at 8 and 11 months. Data on recurrences seem to suggest that the placement of vascular endoprosthesis and nephrostomy, with the simultaneous removal of the ureteral stent and the subsequent break of the mechanic and inflammatory triggers, may led to UAF recovery without the need of surgery [2]. Considering the technological progress of imaging techniques and interventional radiology, we believe that the minimal invasive endovascular approach could be considered the first therapeutic option. Traditional surgery remains the gold standard in case of failure of minimally invasive therapy or if the experience concerning interventional radiology is poor [14]. Ureteral embolization may be useful to support the endovascular procedure in the management of macrohaematuria [13]. We also consider a safer procedure to remove the ureteral stent and to place of a temporary or indwelling nephrostomy and thus to reduce the mechanical damage on the already compromised ureteral wall and to lower inflammation and the risk of urinary infections. The fistula will therefore have the possibility to recover without the need of corrective surgery. However, due to the small sample size of the available studies and the lack of prospective comparative ones in literature, it is not possible to state a definitive recommendation about the best surgical strategy. It is important to underline the essential role of a multidisciplinary appoach including the urologist, the interventional radiologist, and the vascular surgeon in the case of the onset of UAF. As future perspective, two topics should be investigated: (1) the materials of the ureteral stents should be investigated as possible factor favouring UAF development and (2) a detailed histopathological analysis of the ureters and periureteral tissues in chronic stent carriers to improve the knowledge of the UAF etiopathogenesis.

## 5 Conclusions

Ureteral-iliac fistulas can be life threatening for the patient. The medical history plays a key role in the diagnostic and therapeutic process, as it allows to include UAF among the differential diagnoses of gross haematuria. Although our study did not allow us to state definitive conclusion about the diagnostic and therapeutic management of UAF due to small sample size, our findings support previous experiences in favour of the treatment of fistulas with an endovascular approach.
